# Sensorimotor Rehabilitation Using Viscoelastic Material for Upper Limb Recovery after Stroke: A Case Report

**DOI:** 10.1177/11795476261471923

**Published:** 2026-07-23

**Authors:** Inês Agostinho, Ana Temudo, Sérgio Bacelo, Vítor Oliveira, Rafael Bernardes, Luís Sousa

**Affiliations:** 1 59207 Nursing, Universidade Católica Portuguesa, Lisboa, Portugal; 2 679735 Nursing, Unidade Local de Saúde Lisboa Ocidental, Portugal; 3 Nursing, Centre for Interdisciplinary Research in Health (CIIS), Portugal; 4 Faculty of Health Sciences and Nursing (FCSE), Portugal; 5 Higher School of Atlantic Health, Nursing Department, 386390 Atlantic University, Barcarena, Portugal; 6 Comprehensive Health Research Centre, University of Evora, Évora, Portugal

**Keywords:** rehabilitation nursing, stroke, upper extremity, case reports

## Abstract

Upper limb impairment after stroke is a major contributor to functional dependence and reduced quality of life. Although sensorimotor rehabilitation strategies are recommended to promote motor recovery, evidence regarding the therapeutic use of viscoelastic materials remains limited. This case report describes the outcomes of a rehabilitation nursing intervention using a viscoelastic product in a 56-year-old man with multifocal ischemic stroke and left upper limb impairment. The patient completed a 21-day rehabilitation nursing program comprising eight sessions that combined conventional upper limb rehabilitation exercises with sensorimotor stimulation using a viscoelastic product. Functional outcomes were assessed using the Medical Research Council Scale, the Barthel Index, and the Functional Independence Measure. Muscle strength improved from grades 1–3 to grades 4–5 on the Medical Research Council Scale. Functional recovery included improvements in dynamic balance, gait performance, and independence in activities of daily living, with the Barthel Index increasing from 75 (moderate dependence) to 95 (mild dependence). No follow-up data beyond hospital discharge were available to evaluate the long-term maintenance of these functional gains. This case suggests that incorporating a viscoelastic product into a rehabilitation nursing program is feasible and may contribute to improvements in upper limb motor performance and functional independence following stroke. Further prospective studies with larger samples and longer follow-up are warranted.

## Introduction

Stroke and other neurological disorders are among the leading causes of long-term disability worldwide and frequently result in impaired upper limb function, compromising autonomy and performance in activities of daily living.^1,2^ Recovery of upper limb mobility remains one of the main challenges in neurorehabilitation due to the complexity of motor, sensory, and cognitive deficits associated with neurological injury. Rehabilitation nursing (RN) plays an important role in promoting functional recovery through interventions aimed at improving mobility, motor control, and independence.^3^ Conventional rehabilitation approaches commonly include passive and active mobilization exercises, sensory stimulation, antispastic positioning, task-oriented training, self-mobilization techniques, and education regarding adaptive strategies for self-care and prevention of complications associated with immobility. However, given the complexity of neurological recovery, there is an increasing need to explore complementary and innovative therapeutic strategies capable of enhancing sensorimotor integration and patient engagement.^4^

Among emerging approaches, sensorimotor interventions using viscoelastic materials have gained interest due to their tactile and proprioceptive properties. Manipulation of these materials provides multisensory stimulation through texture, resistance, and pressure variations, potentially contributing to motor activation, sensory feedback, attention, and relaxation.^5,6^

Some authors have also associated these sensory experiences with mechanisms related to the Autonomous Sensory Meridian Response, which may influence emotional regulation and concentration through sensory-mediated relaxation responses.^5-7^ Nevertheless, evidence regarding the therapeutic application of viscoelastic materials in neurological rehabilitation remains limited.

This study is grounded in Afaf Meleis’s Transitions Theory, which conceptualizes functional recovery as a dynamic adaptive process involving the acquisition of new skills and responses to health-related changes,^8,9^ triggering a transition experience and, subsequently, active involvement in the process. From this perspective, rehabilitation interventions should support individuals in adapting to functional limitations while promoting active participation in the recovery process.

Although sensorimotor rehabilitation strategies are increasingly used in post-stroke recovery, evidence regarding the therapeutic application of viscoelastic materials remains scarce. Reporting innovative, low-cost, and easily implementable interventions may contribute to expanding the evidence base for rehabilitation nursing practice and generating hypotheses for future controlled studies. Therefore, this case report aimed to describe the outcomes of a rehabilitation nursing intervention using a viscoelastic sensorimotor approach in a person with impaired upper limb mobility following multifocal ischemic stroke.

Therefore, this study aimed to describe the outcomes of a RN intervention using a viscoelastic sensorimotor approach in a person with impaired upper limb mobility following multifocal ischemic stroke.

## Methodology

This article presents a case report on the intervention performed by the RN during a 21-day hospital stay between December 9 to December 30, 2025.

The case concerns a patient with multifocal ischemic stroke and carotid stenosis who underwent carotid endarterectomy; representing a unique clinical case. The person was enrolled in a RN program following the surgical procedure—endarterectomy—on December 17 and subsequent hospitalization, with a total of 8 sessions conducted, each lasting approximately 30 minutes.

Validated assessment instruments recommended for RN practice and validated for the Portuguese population were used to assess functional and motor performance throughout the intervention. Muscle strength was evaluated using the Medical Research Council Scale (MRC), a standardized instrument widely used in neurological rehabilitation to assess voluntary muscle strength. The scale classifies muscle performance on a six-point ordinal scale ranging from 0 to 5, where 0 corresponds to the absence of visible or palpable muscle contraction, 1 to visible contraction without movement, 2 to active movement with gravity eliminated, 3 to active movement against gravity, 4 to active movement against resistance, and 5 to normal muscle strength. This instrument allows monitoring of motor deficits and functional recovery over time.^10^

Functional independence in activities of daily living was assessed using the Barthel Index, an instrument frequently used in stroke rehabilitation to evaluate performance in ten basic activities of daily living, including feeding, bathing, dressing, mobility, transfers, and bladder and bowel control. Total scores range from 0 to 100, with higher scores indicating greater independence. Scores between 0–20 indicate total dependence, 21–60 severe dependence, 61–90 moderate dependence, 91–99 slight dependence, and 100 complete independence. The Portuguese validated version developed by Araújo et al. was used in this study.^11,12^

Functional capacity and level of assistance required were additionally assessed using the Functional Independence Measure (FIM), which evaluates motor and cognitive dimensions through 18 items related to self-care, mobility, communication, and social cognition. Scores range from 18 to 126, with higher values representing greater functional independence.^13^

Balance assessment included observation of static and dynamic balance during sitting and standing activities, according to the RN assessment framework and institutional clinical information system used during hospitalization. This case report was structured in accordance with the CAseREport^14^ guidelines, with necessary adaptations made to account for the specific nature of the clinical case.

It should be noted that the participant signed an informed, clear, and voluntary consent form for health interventions in accordance with Regulation No. 15/2013 of the Directorate-General for Health.

## Case Description

### Clinical History and Initial Assessment

This case report describes a 56-year-old man who was previously independent in activities of daily living. His medical history included smoking (20 pack-years) and daily alcohol consumption (3–6 beers/day). He lived alone and his sister was identified as the primary caregiver.

The patient was admitted to hospital on December 9, 2025, following progressive neurological symptoms that began approximately eight days earlier, including decreased strength in the left upper limb, speech impairment, gait imbalance, and falls. Brain CT angiography revealed ischemic lesions in the territory of the right middle cerebral artery. Doppler ultrasonography identified carotid stenosis of 90% on the left and 50% on the right. A diagnosis of multifocal ischemic stroke involving the right and left middle cerebral arteries was established.

During hospitalization, the patient experienced a fall resulting in transient neurological worsening, with expressive aphasia and confusion. On December 17, 2025, he underwent right carotid endarterectomy with bovine pericardial patch angioplasty under cerebral protection.

### Rehabilitation Nursing Assessment

At admission to the RN program, the patient was conscious, oriented, and motivated for rehabilitation. Mild dysarthria was present, although communication and comprehension were preserved. Functional assessment demonstrated moderate dependence in activities of daily living and impaired mobility, balance, and gait.

Motor assessment using the MRC revealed marked weakness predominantly affecting the left upper limb, particularly wrist and hand function, accompanied by reduced sensory and proprioceptive function. Static and dynamic balance were moderately impaired, and the patient presented a high risk of falls. Cognitive function was preserved.

Based on the RN assessment, the intervention plan focused on muscle movement, balance, gait training, self-care, and preparation for continuity of care. The RN program combined conventional therapeutic exercises with sensorimotor stimulation using a viscoelastic product applied during upper limb exercises (Table 1).

**Table 1.** Focus Areas and Interventions in RN

Nursing diagnosis	Rehabilitation nursing interventions
Moderate impairment of muscle movement	Assessment of muscle strength; passive, active-assisted, and resisted exercises; self-mobilization and positioning exercises; sensorimotor stimulation using a viscoelastic product; strengthening exercises with therapeutic devices according to tolerance.
Moderate impairment of balance	Assessment and training of static and dynamic balance through positioning, postural correction, seated and standing exercises, and trunk control activities.
Moderate difficulty walking	Assessment and training of gait, including assisted walking, hallway and stair training, and encouragement of independent mobility.
Health Services	Assess the need for ongoing care – SOS hours. Refer to long-term care services – SOS hours.
Impaired self-care, moderate degree	Assessment of self-care dependence; education and training regarding assistive devices, adaptive techniques, and home modifications to support activities of daily living.
High potential for regaining autonomy	Encouragement of participation in self-care activities, promotion of independence, motivation, and emotional support throughout the rehabilitation process.

## Results

The RN program consisted of eight sessions conducted over 21 days, with progressive adjustment of exercise complexity according to the patient’s functional evolution.

Functional improvement was observed throughout the intervention period. The Barthel Index score increased from 75 to 95 points, reflecting progression from moderate to mild dependence in activities of daily living (Table 2).

**Table 2.** Assessment of Self-Care Using the Barthel Index

Item/Description	1st session	4th session	8th session
Eating	5	5	5
Transferring	10	15	15
Toilet	5	5	5
Toilet Use	5	10	10
Bathing	5	5	5
Going Up and Down Stairs	5	5	10
Dressing	10	10	10
Bowel Control	10	10	10
Bladder Control	10	10	10
Mobility	10	15	15
Total	75	90	95

Muscle strength improved in both the left upper and lower limbs, with more pronounced gains in shoulder, wrist, and hand function (Table 3).

**Table 3.** Assessment of Muscle Strength Using in the Left Lower Limb Using the Modified MRC

​	1st session	4th session	8th session
Left Lower Limb			
Hip	4	4	5
Knee	4	4	5
Ankle	4	5	5
Left Upper Limb			
Shoulder and elbow	2/3	3/4	5
Wrist and finger abduction	1/2	3/4	4/5
Finger flexion	1/2	3/4	4/5

Dynamic balance improved progressively throughout the rehabilitation program. At the initial assessment, the patient demonstrated impaired dynamic seated balance, while static balance was preserved. By the fourth session, improvements in postural control and trunk stability were observed, with preservation of both static and dynamic balance maintained until the end of the program.

Regarding gait performance, the patient initially presented imbalance during walking, particularly during directional changes and the swing phase of the left lower limb, requiring assistance for safe ambulation. Progressive improvements in gait pattern, stability, and functional mobility were observed throughout the intervention, resulting in independent walking by the final assessment.

## Discussion

The present case report describes the implementation of a RN program incorporating sensorimotor stimulation using a viscoelastic product in a person with upper limb impairment following multifocal ischemic stroke. The findings of this case support the potential role of sensorimotor-based rehabilitation strategies as an adjunct to conventional rehabilitation nursing interventions following stroke. The observed clinical evolution is consistent with current evidence highlighting the importance of combining repetitive motor practice with sensory stimulation to facilitate motor recovery and functional performance.

The progression observed in muscle strength and functional performance is consistent with current evidence supporting repetitive, task-oriented, and sensory-enriched interventions in post-stroke rehabilitation. Task-oriented rehabilitation has been associated with improvements in upper limb recovery, motor control, and functional performance after stroke. Recent systematic reviews suggest that repetitive and meaningful motor practice may promote neuroplasticity and improve upper extremity function, particularly when interventions are individualized and progressively adjusted to the person’s abilities.^15^

Similarly, sensory-based rehabilitation approaches have gained increasing attention in neurological rehabilitation due to their potential contribution to sensorimotor integration and cortical reorganization. Somatosensory stimulation strategies, including repetitive sensory stimulation and somatosensory retraining, have been associated with improvements in somatosensory discrimination, balance, and upper limb performance in some stroke populations, although findings remain heterogeneous across studies.^16,17^ In the present case, the manipulation of a viscoelastic material may have enhanced tactile and proprioceptive input during therapeutic exercises, potentially contributing to increased engagement during the rehabilitation sessions. The observed improvements in balance, gait, and activities of daily living are also aligned with previous stroke rehabilitation studies demonstrating the benefits of progressive therapeutic exercise and sensorimotor interventions. Evidence suggests that task-oriented rehabilitation programs may contribute to improvements in gait performance, postural control, and functional independence after stroke.^15^ Recovery of independence in activities of daily living remains one of the principal goals of stroke rehabilitation and is strongly associated with upper limb recovery and mobility gains. The viscoelastic product used in this intervention may have acted as an adjunctive therapeutic resource by providing continuous tactile and proprioceptive stimulation during movement execution. Integration of visual, tactile, and proprioceptive inputs is recognized as an important component of neuromotor recovery following stroke. Studies examining peripheral somatosensory stimulation have reported potential benefits in motor recovery, postural control, and upper limb function, particularly when sensory stimulation is combined with intensive motor training.^18,19^ Furthermore, recent studies investigating tactile stimulation approaches suggest that sensory stimulation may contribute to sensory-motor recovery and increased participation during rehabilitation interventions.^20^

From a clinical perspective, the intervention described in this case appears feasible, low-cost, and easily integrated into RN practice. The use of simple sensorimotor resources may represent an accessible complement to conventional rehabilitation strategies, particularly in settings with limited technological or financial resources.

Nevertheless, several limitations should be considered when interpreting these findings. First, this study describes a single clinical case, which limits generalizability to broader stroke populations. Second, the absence of a control condition prevents determination of the specific contribution of the viscoelastic intervention, since the observed improvements may also reflect spontaneous neurological recovery, conventional rehabilitation exercises, or multidisciplinary care. Third, the intervention period was relatively short and no long-term follow-up was conducted to assess maintenance of functional gains over time. Additionally, some outcomes relied on clinical assessment instruments and observational evaluation, which may be influenced by evaluator interpretation. Current evidence regarding somatosensory interventions after stroke also remains heterogeneous, with variability in intervention protocols, treatment intensity, and outcome measures.^21^

Follow-up data beyond hospital discharge were not available, preventing evaluation of the long-term maintenance of the observed functional gains.

## Conclusion

This case report describes the clinical course of a patient diagnosed with multifocal ischemic stroke over the course of 21day period of RN intervention, comprising 8 sessions. The results suggest progressive improvement in muscle strength, particularly in the left upper limb, as well as recovery in balance, gait, and independence in activities of daily living.

The integration of a sensorimotor intervention using a viscoelastic product is clinically feasible, safe, and potentially conducive to functional recovery, constituting a relevant complement to conventional RN approaches.

This study reinforces the role of the RN in implementing innovative interventions in the health-disease care transition process, contributing to the maximization of healthoutcomes.

Further studies using controlled methodologies, larger samples, and longer follow-up periods are needed to better clarify the therapeutic contribution of viscoelastic sensorimotor interventions in post-stroke rehabilitation.

## Implications for Practice

This case report suggests that innovative approaches in RN practice, when used in conjunction with conventional interventions for individuals with neurological conditions, may provide additional benefits. The role of the RN is essential in the comprehensive assessment of functionality, the identification of motor and sensory deficits, and the implementation of individualized rehabilitation programs.

## Data Availability

All data pertinent to this case report have been included in this article. Further inquiries can be directed to the corresponding author.*

## Appendix

### Abbreviations

FIM: Functional Independence Measures
MRC: Medical Research Council Scale
RN: Rehabilitation Nursing
